# The emerging role of viral vectors as vehicles for *DMD* gene editing

**DOI:** 10.1186/s13073-016-0316-x

**Published:** 2016-05-23

**Authors:** Ignazio Maggio, Xiaoyu Chen, Manuel A. F. V. Gonçalves

**Affiliations:** Department of Molecular Cell Biology, Leiden University Medical Center, Einthovenweg 20, 2333 ZC Leiden, The Netherlands

## Abstract

Duchenne muscular dystrophy (DMD) is a genetic disorder caused by mutations in the dystrophin-encoding *DMD* gene. The *DMD* gene, spanning over 2.4 megabases along the short arm of the X chromosome (Xp21.2), is the largest genetic locus known in the human genome. The size of *DMD*, combined with the complexity of the DMD phenotype and the extent of the affected tissues, begs for the development of novel, ideally complementary, therapeutic approaches. Genome editing based on the delivery of sequence-specific programmable nucleases into dystrophin-defective cells has recently enriched the portfolio of potential therapies under investigation. Experiments involving different programmable nuclease platforms and target cell types have established that the application of genome-editing principles to the targeted manipulation of defective *DMD* loci can result in the rescue of dystrophin protein synthesis in gene-edited cells. Looking towards translation into the clinic, these proof-of-principle experiments have been swiftly followed by the conversion of well-established viral vector systems into delivery agents for *DMD* editing. These gene-editing tools consist of zinc-finger nucleases (ZFNs), engineered homing endoculeases (HEs), transcription activator-like effector nucleases (TALENs), and RNA-guided nucleases (RGNs) based on clustered, regularly interspaced, short palindromic repeats (CRISPR)–Cas9 systems. Here, we succinctly review these fast-paced developments and technologies, highlighting their relative merits and potential bottlenecks, when used as part of in vivo and ex vivo gene-editing strategies.

## Background

Duchenne muscular dystrophy (DMD) is a lethal X-linked genetic disorder (affecting approximately 1 in 5000 boys) [1] caused by mutations in the ~2.4-megabase *DMD* gene [2] which lead to irrevocable muscle wasting owing to the absence of dystrophin in the striated muscle cell lineage [3]. Although dystrophin-disrupting mutations can be of different types, 68 % of them consist of intragenic large deletions [4]. These deletions can be found along the entire length of the enormous *DMD* locus, with 66 % nested within a major, recombination-prone, hotspot region spanning exons 45 through 55 [4]. The resulting joining of exons flanking DMD-causing mutations by pre-mRNA splicing yields transcripts harboring out-of-frame sequences and premature stop codons, which are presumably degraded by nonsense-mediated mRNA decay mechanisms.

In muscle cells, the long rod-shaped dystrophin protein anchors the intracellular cytoskeleton to the extracellular matrix via a large glycoprotein complex embedded in the plasma membrane called the dystrophin-associated glycoprotein complex (DGC). This structural link is fundamental for proper cellular signaling and structural integrity. Indeed, in the absence of dystrophin, a relentless degenerative process is initiated that consists of the substitution of muscle mass by dysfunctional fibrotic and fat tissues [3]. As time elapses, patients with DMD become dependent on a wheelchair for ambulation and, later on, require breathing assistance. Crucially, with the aid of palliative treatments, which include supportive respiratory and cardiac care, the life expectancy of patients with DMD is improving and a greater proportion of these patients now reach their late 30s [5].

## Targeting the root cause of DMD

The complexity of DMD, combined with the extent of affected tissue, demands the development of different, ideally complementary, therapeutic approaches. The goal of pursuing parallel approaches is to target different aspects and stages of the disease and hence maximize the length and quality of patients’ lives. Towards this end, various candidate therapies are currently under intense investigation [3, 5, 6]. These research lines include: (1) mutation-specific exon skipping via modulation of pre-mRNA splicing by antisense oligonucleotides; (2) compensatory upregulation of dystrophin’s autosomal paralog utrophin by small-molecule drugs or artificial transcription factors; (3) cell therapies involving allogenic myogenic stem/progenitor cell transplantation; and (4) gene therapies based on the delivery of shortened versions of dystrophin (for example, microdystrophins) to affected tissues. Of note, these recombinant microdystrophins are devoid of centrally located motifs that are, to some extent, dispensable. The miniaturization bypasses the fact that the full-length 11-kilobase (kb) dystrophin coding sequence is well over the packaging limit of most viral vector systems.

**Fig. 1:**
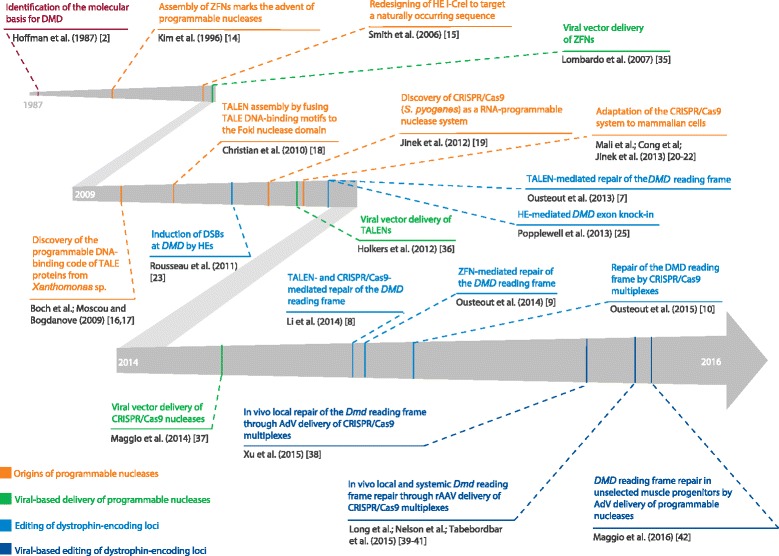
Milestones on the path towards somatic genetic therapies for Duchenne muscular dystrophy that rely on viral-based *DMD* editing. The time marks correspond to the first release date of the referenced articles (for example, advanced online publication). *AdV* adenoviral vector, *CRISPR–Cas9* clustered regularly interspaced short palindromic repeat-associated Cas9 nuclease, *DMD* Duchenne muscular dystrophy, *DSB* double-stranded DNA break, *HE* homing endonuclease, *rAAV* recombinant adeno-associated virus, *TALE* transcription activator-like effector

More recently, genome-editing strategies based on sequence-specific programmable nucleases have been proposed as another group of therapies for DMD [7–10]. Programmable nucleases are tailored to induce double-stranded DNA breaks (DSBs) at predefined positions within complex genomes [11–13]. In chronological order of appearance, these enzymes are: zinc-finger nucleases (ZFNs) [14], engineered homing endonucleases (HEs) [15], transcription activator-like effector nucleases (TALENs) [16–18], and RNA-guided nucleases (RGNs) based on dual RNA-programmable clustered, regularly interspaced, short palindromic repeat (CRISPR)–Cas9 systems [19–22] (Fig. 1). HEs, also known as meganucleases, from the LAGLIDADG family can be engineered to cleave DNA sequences other than those of their natural target sites. The designing of new substrate specificities depends, however, on complex protein engineering efforts involving the screening of large combinatorial assemblies of HE parts [15]. Regardless, redesigned HE were shown to create indel footprints at intronic DMD sequences, albeit at very low frequencies (<1 % of target alleles in human myoblasts) [23]. In contrast to the construction of redesigned HEs, the modular nature of the DNA-binding motifs of ZFNs and TALENs makes them more amenable to protein engineering [14, 16–18]. Of note, the assembly of highly specific TALENs is particularly straightforward owing to a simple one-to-one relationship between the binding of each of their DNA-binding modules, that is, transcription activator-like effectors (TALE) repeats, and a specific nucleotide [16, 17]. Among other features, ZFNs and TALENs differ from RGNs in that they are chimeric enzymes that assemble at their target nucleotide sequences as catalytically active dimers through protein–DNA binding, whereas RGNs are ribonucleoprotein complexes whose DNA cutting specificities are ultimately governed by DNA–RNA hybridization. Indeed, RGNs consist of a Cas9 endonuclease and a sequence-customizable single-guide RNA (sgRNA) moiety that addresses the protein component to a specific target site. Typically, the target site consists of 18–20 nucleotides complementary to the 5′ end of the sgRNA and a protospacer adjacent motif (PAM; NGG and NNGRRT in the case of the prototypic *Streptococcus pyogenes* Cas9 and its smaller orthologue *Staphylococcus aureus* Cas9, respectively) [19, 24]. Hence, in comparison with the strictly protein-based systems, RGNs are more versatile owing to their mode of construction, which does not involve protein engineering [11–13].

Regardless of the DNA cutting system that is selected, the repair of the ensuing DSBs by different endogenous cellular DNA repair processes can yield specific genome editing outcomes. For example, the engagement of homologous recombination (HR) and non-homologous end joining (NHEJ) mechanisms can result in targeted exogenous DNA additions and endogenous DNA deletions, respectively [11–13]. The incorporation of small insertions and deletions (indels) following the repair of DSBs by NHEJ can also be exploited for knocking out *trans*-acting and *cis*-acting genomic elements [11–13]. By operating at the DNA level, such interventions can potentially lead to the correction of disease-causing mutations on a permanent basis.

## *DMD* gene editing

*DMD* editing based on targeted addition of “exon patches” corresponding to missing or disrupted coding sequences might become ideal therapeutic options as they result in the synthesis of full-length dystrophin [8, 25]. Proof-of-principle experiments demonstrated that combining *DMD*-repairing exon patches with engineered meganucleases [25], RGNs, or TALENs [8] can indeed restore full-length message coding for dystrophin. At present, however, most *DMD* editing approaches under investigation are based on inducing NHEJ to disrupt or delete specific sequences [7–10]. These strategies exploit the fact that, in contrast to HR, NHEJ is active in both dividing and post-mitotic cells [26, 27], which makes these approaches more amenable to both ex vivo and in vivo applications (Table 1). The NHEJ-based strategies also capitalize on the fact that internally truncated in-frame *DMD* transcripts, despite being shorter than the full-length *DMD* transcript, often yield functional dystrophins [28–30]. Indeed, such dystrophins are characteristic of patients with Becker muscular dystrophy, whose disease phenotypes are milder than those of their counterparts with DMD [28–30]. Therefore, programmable nucleases have been tailored for correcting defective *DMD* alleles by targeting: (1) splicing sites for inducing DNA-borne exon skipping; (2) exonic sequences for resetting reading frames and “overwriting” downstream premature stop codons; and (3) flanking intronic sequences for directly excising mutations through the use of pairs of programmable nucleases (multiplexing) [7–10]. DNA-borne exon skipping by NHEJ-mediated splicing motif knockout and reading-frame resetting by frame shifting are mutation-specific and rely on the fraction of indel footprints that yield in-frame sequences. Importantly, the resulting indels might introduce immunogenic epitopes into de novo-synthesized dystrophin molecules. Depending on certain variables (for example, revertant mutation backgrounds), these epitopes might be recognized as foreign by the immune system. Related to this potential issue, T-cell immunity to dystrophin epitopes was detected in two patients undergoing a clinical trial based on recombinant adeno-associated viral vector (rAAV) delivery of a microdystrophin construct [31].

**Table 1:** Comparison of ex vivo and in vivo viral-based *DMD* editing strategies under investigation

✓ Pros × Cons	Viral-based *DMD* editing	
	Ex vivo	In vivo
Background	× Knowledge about the grafting of different types of myogenic cells into recipient human muscles is scarce	✓ Builds upon an increasing amount of knowledge about the in vivo administration of viral vectors into recipient human muscles (for example, microdystrophin-encoding rAAVs)
Production	✓ Potentially less dependent on large-scale production of viral vectors × Reliant on the upscaling of cell culture systems × The required numbers of certain myogenic cell types might not be achievable owing to senescence (for example, myoblasts) × The current protocols do not permit culturing bona fide skeletal muscle stem cells (that is, satellite cells) in vitro	✓ Independent from the upscaling of cell culture systems × More reliant on large-scale production of viral vectors
Delivery	✓ Well-defined genetic modification environment that enables careful monitoring of procedures, events, and outcomes ✓ Lower stringency for monitoring the biodistribution (for example, gonads and shedding of vector elements) × Protocols for effective myogenic cell engraftment, migration, and differentiation need to be improved (for example, via signaling gradients and cell-autonomous reprogramming of iPSCs) × Local and locoregional administration of myogenic cells might be difficult to apply to a broad range of muscle groups × Protocols for the systemic delivery and tissue homing of myogenic cells need to be developed	✓ Direct exposure to gene-editing tools facilitates in situ correction of differentiated striated muscle tissues ✓ Possible in situ transduction of resident tissue-specific stem cells might generate a long-term source of gene-edited muscle progenitor cells ✓ Expanding range of viral vector pseudotypes enables the investigation of different transduction patterns—for example, tropism for affected tissues—while avoiding APCs. Such transductional targeting can be combined with transcriptional targeting (that is, use of tissue-specific promoters) × Local and locoregional administration of viral vector particles might be difficult to apply to a broad range of muscle groups × Protocols for the systemic delivery of viral vectors to affected tissues need to be improved Higher stringency for monitoring the biodistribution (for example, gonads and shedding of vector elements)
Strategy	✓ Relies mostly on targeting replicating cells that are amenable to gene-editing approaches based on NHEJ as well as HR	× Relies mostly on targeting post-mitotic cells, which are less amenable to HR-based gene-editing principles
Immunology	✓ Minimizes the exposure of the patient to immunogenic components of viral vectors and gene-editing tools ✓ Possibly compatible with the re-administration of gene-edited autologous cells ✓ Avoids the blocking of viral vector particles by neutralizing antibodies present in the majority of the human population	× Patient exposure to immunogenic components of vector particles and gene-editing tools. Possible mounting of cellular responses to transduced cells displaying foreign epitopes × Anti-vector neutralizing antibodies in the majority of the human population. Serotype cross-neutralizing activity might render vector pseudotyping and vector re-administration ineffective

In vivo approaches entail the direct administration of gene-editing viral vectors to the patient. Ex vivo approaches encompass the in vitro transduction of patient-derived cells (for example, myogenic stem or progenitor cells) with gene-editing viral vectors, which is followed by cell culture and autologous transplantation back into the patient. Both treatment modalities can, in principle, be applied either locally or systemically. *APCs* antigen-presenting cells, *HR* homologous recombination, *iPSCs* induced pluripotent stem cells, *NHEJ* non-homologous end joining, *rAAVs* recombinant adeno-associated viruses

In contrast to those triggering single-exon deletions, the *DMD* correction approaches based on targeted multi-exon deletions do not give rise to indel-derived epitopes and are applicable to a wider range of DMD-causing genotypes, with de novo-generated intronic junctions leading to predictable in-frame mRNA templates [10, 32]. However, multiplexing approaches carry increased risks for unwarranted, possibly deleterious, genome-modifying events (for example, off-target DSBs, inversions, and translocations), owing to their dependency on two programmable nucleases rather than one [12]. These increased risks will be present despite the fact that targeted DSBs in boys with DMD will be restricted to a single allele.

## Viral-based *DMD* editing

The clinical application of *DMD*-editing concepts will require improved methods for delivering large and complex molecular tools into target cells, as well as increasing the efficiency, specificity, and fidelity of the ensuing DNA modifications [12]. Similarly to their effective contribution to “classic” gene replacement therapies [33], viral vectors are expected to become instrumental tools for investigating and developing therapeutic gene-editing approaches ex vivo and in vivo (for a recent review on the adaptation and testing of viral vector systems for genome editing purposes, see [34]). Indeed, ZFNs, TALENs, and RGNs have all been shown to be amenable to viral vector delivery [35–37] (Fig. 1). More recently, adenoviral vectors (AdVs) and rAAVs have been successfully converted into *DMD*-editing agents in both patient-derived cells and mouse models of DMD [38–42] (Fig. 1).

### In vivo

The *Dmd*^*mdx*^ mouse model has a (mild) dystrophic phenotype that is due to a nonsense mutation located in exon 23 of the *Dmd* gene; historically, this has been the principal animal model for investigating DMD-targeted therapies and certain pathophysiological aspects of the disease [43]. In one study, conventional, commonly used, serotype-5 AdVs constructed to encode either *S. pyogenes* Cas9 or sgRNAs that targeted sequences flanking *Dmd* exons 21 through 23 were co-injected into the gastrocnemius muscles of newborn *Dmd*^*mdx*^ mice [38]. At 3 weeks post-injection, dystrophin synthesis was readily detected in transduced muscle fibers. A semi-quantitative assay based on western blot analysis estimated that these fibers contained ~50 % of the wild-type levels of dystrophin. The gene-edited muscle regions displayed reduced Evans blue dye uptake under rest and force-generating conditions, indicating improved muscle fiber integrity.

A notorious characteristic of prototypic serotype-5 AdVs is their immunogenicity and, although they can be made without viral genes [34, 44], capsid-cell interactions can still trigger strong innate immune responses [45, 46]. In addition, the high prevalence of neutralizing antibodies directed against the capsids of serotype-5 AdVs in the human population has contributed to spurring the development of AdVs based on alternative serotypes [45]. Historically, these immunological determinants have in fact precluded the efficacious deployment of AdV technologies in “classic” gene therapy settings in which long-term maintenance of transduced cells is a prerequisite. AdVs are currently mostly used in human individuals either as oncolytic or vaccination agents [47]. The use of AdVs in translational in vivo gene editing will require dampening their immunogenicity and improving their targeting to specific cell types or organs. These efforts will be heavily guided by insights into the biology of host–vector interactions [45, 46]. For example, while serotype-5 AdVs bind through their fibers to the coxsackievirus and adenovirus receptor (CAR) to enter cells in vitro [48], their uptake by liver cells after intravenous administration in vivo is CAR-independent and governed by the interaction of their hexons with blood coagulation factors [49].

Three other studies investigated the in vivo delivery of RGN components (that is, sgRNA and Cas9 nucleases) by capsid-pseudotyped rAAVs for inducing the in-frame deletion of *Dmd* exon 23. These rAAV particles consist of rAAV DNA from serotype 2 packaged in capsids from AAV serotype 8 (rAAV-8) [40] or serotype 9 (rAAV-9) [39, 41], whose tropism for striated mouse muscle had previously been established [50, 51]. Pairs of these vectors encoding sgRNAs and either *S. pyogenes* Cas9 [39] or the smaller *S. aureus* Cas9 [40, 41] were co-administered into newborn and adult *Dmd*^*mdx*^ mice. Nelson and colleagues detected abundant dystrophin protein synthesis 8 weeks after co-injecting a mixture of rAAV-8 particles encoding *S. aureus* Cas9 and cognate sgRNAs into tibialis anterior muscles [40]. Importantly, treated muscles had improved contractibility and force-generating functions. Finally, by capitalizing on the well-established performance of rAAV-8 after systemic administration in mice [50], Nelson and colleagues were able to detect dystrophin in cardiac muscle tissue after a single intravenous injection [40].

Instead of rAAV-8, Long and colleagues used rAAV-9 to introduce *S. pyogenes* RGN complexes into striated muscle tissues of newborn *Dmd*^*mdx*^ mice [39]. Dystrophin was detected in striated muscle tissues after local and systemic administration of the engineered viral vectors [39]. Consistent with the slow kinetics of gene expression from rAAVs, which might in part be related to the processes underlying the conversion of vector DNA from a single-stranded to a transcriptionally active double-stranded form [52], a time-dependent increase in dystrophin buildup was observed. For instance, tibialis anterior muscles of postnatal day 12 *Dmd*^*mdx*^ mice subjected to direct intramuscular injections with the engineered viral vector contained approximately 8 and 26 % of dystrophin-positive fibers at 3 and 6 weeks post-administration, respectively [39].

In the third study, Tabebordbar and coworkers used rAAV-9 pairs for delivering *S. aureus* Cas9 and sgRNAs to the tibialis anterior muscle of dystrophin-defective *Dmd*^*mdx*^ mice [41]. Similarly to the results of the two other studies on rAAV-mediated *Dmd* exon 23 deletion experiments [39, 40], administration of the rAAV-9 pairs led to robust rescue of dystrophin protein synthesis in transduced muscles and to a concomitant measurable improvement in functional parameters (that is, specific force and force drop) compared with those in unedited controls [41]. In addition, intraperitoneal co-injection of rAAV-9 particles into dystrophic mice led to frequencies of *Dmd* exon 23 excision in cardiac and skeletal muscle tissues ranging from 3 to 18 %, as determined by RT-PCR, depending on the muscle groups analyzed [41]. Importantly, *Dmd*-editing rAAV-9 particles were also administered intramuscularly or systemically to *Pax7-ZsGreen Dmd*^*mdx*^ mice whose satellite cells are marked by green fluorescence. Subsequently, after isolating, expanding, and inducing myogenic differentiation of the Pax7-ZsGreen-positive cells, the authors reported in-frame *Dmd* exon 23 deletions in myotubes derived from these cells [41]. The population of Pax7-positive satellite cells harbors the resident mononuclear stem cell population of skeletal muscle and is typically lodged between the sarcolemma of muscle fibers and the basal lamina [53]. The “stemness” qualities of self-renewal and lifelong differentiation capacity make these tissue-specific stem cells ideal substrates for regenerative medicine approaches for treating muscular dystrophies as, in contrast to their committed progenitor offspring, these cells support robust long-term tissue homeostasis and repair [54, 55]. Recent experiments in transgenic *Dmd*^*mdx*^ mice showed that, in addition to its other functions, dystrophin has a transient but critical regulatory role in activated Pax7-positive satellite cells, which further supports the therapeutic relevance of this cell population. In particular, the 427-kilodalton dystrophin isoform is expressed at very high levels in these cells, where it governs asymmetric cell division, a process that is indispensable for maintaining the stem cell pool and for generating committed Myf5-positive myoblast progenitors for muscle repair [56]. Among other processes, this mechanism presumably involves interactions between the spectrin-like repeats R8 and R9 of dystrophin and Mark2, a protein that regulates cell polarity [56, 57]. If conserved in humans, this cell-autonomous mechanism would be evidence that DMD is also a stem cell disease, which would strengthen the view that satellite cells should be preferential targets for DMD therapies, in addition to differentiated cells. Interestingly, the very high amounts of dystrophin seen in activated Pax7-positive satellite cells are followed by very low and intermediate levels of the protein in myoblasts and differentiated muscle cells, respectively [56]. Such differentiation-stage-specific oscillations in dystrophin amounts strengthen the rationale for repairing the genetic defects by direct endogenous *DMD* editing, as this strategy is expected to restore proper regulation of dystrophin synthesis.

Taken together, these findings demonstrate that rAAV delivery of RGN complexes can result in the structural improvement of treated striated tissues and also lead to the partial rescue of specific muscle functions in dystrophic mice. Although dystrophin synthesis was detected at 6 months after a single injection in one experiment [40], no long-term detailed assessments of these approaches were done. Regardless, the available data do support the potential of these vectors as in vivo *DMD*-repairing agents, thus warranting further research. Future developments should include assuring the transient presence of programmable nucleases in post-mitotic tissues, preclinical testing in large outbred animal models [43], and identifying or engineering rAAV capsids that have preferential tropism for human striated muscle cells, including satellite cells, while bypassing the host’s humoral immunity against prevalent AAV serotypes [58].

The administration of rAAVs to some human individuals resulted in clinical endpoints that had not been predicted on the basis of the available preclinical data. These findings are simultaneously sobering and illuminating. An example is provided by the elimination of transduced hepatocytes in patients with hemophilia B, which was due to the development of a dose-dependent T-cell response to capsid epitopes from an rAAV-2-encoding human factor IX [59]. This type of dose-dependent cellular immune response has also been documented in human skeletal muscle cells transduced with rAAVs [60], although it is of note that the emergence of T-cell responses directed against rAAV capsid epitopes does not always equate with the elimination of transduced muscle cells [61]. In addition, short-term immune suppression might help to dampen cellular immune responses in muscular dystrophy patients subjected to high-doses of rAAV particles [62]. It is worth mentioning, however, that the altered immune cell composition and inflammatory environment that characterize dystrophic muscle tissue might introduce potential confounding factors associated with in vivo rAAV delivery. Knowledge about these issues and preclinical data obtained from canine models of DMD [63–65] are guiding the design of new clinical trials based on the administration of rAAVs to patients with DMD [66]. Further insights are being gathered from the application of rAAVs to patients suffering from other muscular disorders such as Limb-girdle muscular dystrophy caused by α-sarcoglycan deficiency [67]. In particular, there is mounting evidence for the importance of restricting transgene expression to muscle cells by using tissue-specific promoters [67]. In the future, muscle-restricted transgene expression might be further improved by combining transcriptional with transductional targeting through rAAVs with capsids with a strict tropism for human muscle tissue. The recently discovered pan-AAV receptor AAVR [68] is likely to have an important role in this research; for instance, by shedding light on rAAV transduction profiles in different cell types, including immune-related cells. Therefore, although rAAVs have a substantially milder immunogenic profile than that of AdVs, they also need to be adapted for translational in vivo gene-editing purposes, which, as for AdVs, will be rooted in an increasing knowledge about vector-host interactions and biodistribution at the organismal level. Finally, in the context of future clinical protocols for in vivo *DMD* editing, the synthesis of programmable nucleases should be restricted not only spatially but also temporally.

### Ex vivo

Ex vivo *DMD* editing strategies to generate genetically corrected human cells with myoregenerative capacity for autologous transplantation can also be envisaged (Table 1). These approaches offer a controlled genome-modification environment, bypass vector-neutralizing antibodies, and minimize direct contact between the patient and immunogenic components, such as those from vector particles, gene-editing tools, and allogenic donor cells (Table 1). Importantly, provided that clinically applicable delivery vehicles of gene-editing tools become available, ex vivo *DMD* editing can naturally build upon the numerous investigations that are being conducted on the isolation, characterization, and testing of human myogenic cells isolated from different tissues for treating muscular dystrophies [69–73]. These cellular substrates include satellite cells [53, 54] and their committed myoblast progeny [74], induced pluripotent stem cells [75], mesenchymal stromal cells [76, 77], vasculature-associated mesoangioblasts/pericytes [78], and blood-derived CD133^+^ cells [79]. Of note, the latter two cell types have been shown to be amenable to systemic administration in animal models and, to some extent, can colonize their satellite cell niche [80–82]. In addition, mesoangioblasts/pericytes and CD133^+^ cells have entered early stage clinical testing in the context of allogenic cell therapies for DMD [83, 84]. These clinical investigations complement earlier and ongoing testing of allogenic myoblast transplantation that are based on intramuscular injections [71–73, 85, 86].

Despite these encouraging developments, the hurdles towards the clinical application of ex vivo DMD cell therapies remain numerous and complex. Preeminent examples of such hurdles include achieving sufficient numbers of undifferentiated cells in vitro, as well as robust cell engraftment, migration, and differentiation of the transplanted graphs in vivo. Ideally, the transplanted cells should also be capable of homing to damaged tissue after systemic administration and should dedifferentiate or transdifferentiate (when belonging to muscle and non-muscle lineages, respectively) into satellite cells (Table 1). Therefore, although certain therapeutic-cell candidates are well positioned to fulfil some of these criteria, none of them fulfils all of the criteria yet [69, 72]. For example, CD133^+^ blood-derived cells and mesoangioblasts/pericytes have been shown to be compatible with systemic administration procedures in preclinical models of muscular dystrophies [78, 79] but their contribution to effective myoregeneration requires further investigation. In contrast, the features of human satellite cells make them natural, highly potent, muscle-repairing entities. Besides being available in diverse human muscle groups, satellite cells have the capacity to readily engraft as functional stem cells and robustly contribute to de novo muscle repair in xenotransplantation experiments [72]. However, harvested satellite cells are not amenable to systemic administration or current ex vivo culture conditions, as they readily differentiate into myoblasts with reduced regenerative capacity [87]. Importantly, the latter hurdle might not be insurmountable, as ongoing research indicates that extrinsic factors such as the composition and elasticity of culture vessels can be modulated to mimic the rigidity of the native satellite cell niche (that is, 12 instead of ~10^6^ kilopascals) and, in doing so, enable the in vitro survival and self-renewal of bona fide satellite cells [88]. The development of such biomimetic tissue-engineering technologies directed to the in vitro expansion of human satellite cells is in demand.

In addition to that of skeletal muscle, cardiac muscle impairment is a key component of DMD that also needs to be tackled in future therapies. Despite intense research on the isolation and characterization of stem and progenitor cells for the repair of damaged heart tissue (for example, after ischemia), so far there is no evidence for a significant functional improvement of the myocardium through the cell-autonomous differentiation of the transplanted cells into mature, electrically coupled cardiomyocytes [89, 90].

Other equally important areas for further research in the field of DMD-targeted regenerative medicine are: (1) deepening our knowledge about the origins and biology of the various cell therapy candidates and their interaction(s) with their respective niches; (2) gathering all possible information on the fate and behavior of transplanted cells from ongoing and future cell therapy trials; (3) moving forward with gene replacement approaches involving stable transduction of recombinant constructs; and (4) testing different gene-editing reagents and strategies for developing autologous cell transplantation approaches. Regarding the latter research avenue, it will be crucial to efficiently introduce different gene-editing tools into human muscle progenitor cells and non-muscle cells with myogenic capacity. AdVs outperform rAAVs in ex vivo settings owing to their higher functional vector particle titers, larger packaging capacity (up to 37 kb), and faster kinetics of transgene expression [34, 52]. Our laboratory has recently reported that tropism-modified AdVs are particularly efficient and versatile vehicles for introducing RGNs and TALENs into CAR-negative myoblasts from patients with DMD [42]. The strict episomal nature of the transduced AdV genomes enabled transient high-level expression of programmable nucleases that corrected native *DMD* alleles and yielded permanent and regulated dystrophin synthesis. In this work, we exploited targeted NHEJ-mediated correction of DMD-causing intragenic deletions by reading-frame resetting, DNA-borne exon skipping, and in-frame excision of single or multiple exons [42]. The rescue of dystrophin synthesis could be readily detected in unselected populations of target cells [42]. Bypassing the need for cell selection expedients is expected to simplify and help translate ex vivo DMD editing protocols to the clinic. Moreover, AdV-based delivery systems will aid with assessing and comparing different *DMD* editing reagents and strategies in panels of human myogenic cells harboring the various *DMD* mutations, which are not represented in the currently available animal models. In addition, the well-defined in vitro conditions permit the straightforward monitoring of intended as well as unwarranted or potentially deleterious interactions between the gene-editing reagents and the human genome (Table 1). Prominent examples of such quality controls will include the genome-wide tracking of adverse DNA-modifying events directly in patient cells (for example, off-target activities of programmable nucleases).

## Conclusions and future directions

The application of genome-editing principles for *DMD* repair purposes is expanding the range of genetic therapy options for tackling DMD. In this context, the coopting of viral vector systems as carriers of programmable nucleases is set to have an important role in the path to DNA-targeted DMD therapies and, along the way, in defining the best strategies and optimizing the corresponding reagents. In view of the complexity of the DMD phenotype and the extent of the affected tissues, it is sensible to consider that future DMD therapies will profit from integrating complementary approaches. For example, the simultaneous treatment of skeletal and cardiac tissues from patients with DMD might be approached by combining ex vivo and in vivo gene-editing strategies, respectively. Such schemes can potentially address the skeletal and heart components of DMD while circumventing the current lack of cell entities capable of differentiating into functional cardiomyocytes. Regardless of the particular therapy or combination of therapies ultimately selected, there is widespread agreement that they should preferably be applied as early as possible so that most striated musculature is still in place and the degeneration process can be halted or, ideally, reversed in the treated muscle groups. Finally, the insights gained from these DMD-targeted research efforts will probably also be useful for devising advanced genetic therapies for addressing other neuromuscular disorders for which, at present, there are no therapeutic options available.

## Abbreviations

AdV: adenoviral vector
APC: antigen-presenting cell
CAR: coxsackievirus and adenovirus receptor
CRISPR: clustered, regularly interspaced, short palindromic repeats
DGC: dystrophin-associated glycoprotein complex
DMD: Duchenne muscular dystrophy
DSB: double-stranded DNA break
HR: homologous recombination
indel: insertion and deletion
iPSC: induced pluripotent stem cell
kb: kilobase
NHEJ: non-homologous end joining
PAM: protospacer adjacent motif
rAAV: recombinant adeno-associated viral vector
RGN: RNA-guided nuclease
sgRNA: single-guide RNA
TALE: transcription activator-like effector
TALEN: transcription activator effector-like nuclease
ZFN: zinc-finger nuclease
